# Knowledge-Based Remote E-Coaching Framework Using IoT Devices for In-Home ADL Rehabilitation Treatment of Degenerative Brain Disease Patients

**DOI:** 10.3390/s22207957

**Published:** 2022-10-19

**Authors:** Hyo-Jung Kim, Seol-Young Jeong, Soon-Ju Kang

**Affiliations:** 1 School of Electronic and Electrical Engineering, Kyungpook National University, Daegu 41566, Korea; 2 Software Education Institute, Kyungpook National University, Daegu 41566, Korea

**Keywords:** knowledge-based system, activities of daily living (ADL), e-coaching framework, user behavior recognition, IoT devices

## Abstract

The activities of daily living (ADL) ability level of an elderly patient is an important indicator in determining the patient’s degree of degenerative brain disease and is mainly evaluated through face-to-face interviews with doctors and patients in hospitals. It is impossible to determine the exact ADL ability of a patient through such a temporary interview, and the pursuit of accurate ADL ability evaluation technology is a very important research task worldwide. In this paper, in order to overcome the limitations of the existing ADL evaluation method mentioned above, first of all, a self-organized IoT architecture in which IoT devices autonomously and non-invasively measure a patient’s ADL ability within the context of the patient’s daily living place was designed and implemented. Second, a remote rehabilitation treatment concept for enhancing the patient’s ADL ability we call an “e-coaching framework”, in which a doctor remotely gives an instruction in a specific ADL scenario, and the patient’s ability to understand and perform the instruction can be measured on-line and in real time, was additionally developed on top of the self-organized IoT architecture. In order to verify the possibility of remote rehabilitation treatment through the proposed architecture, various remotely directed ADL scenarios were performed and the accuracy of the measurements was verified.

## 1. Introduction

If it is possible to remotely rehabilitate patients with degenerative brain disease who are not able to move freely, it will prove to be a very important and effective solution in the current COVID-19 pandemic era. In order for this technology to become possible, a technology that accurately identifies a patient’s daily living ability at home must be developed.

Activities of daily living (ADL) refers to the abilities essential for daily life, such as moving, cooking, and using tools [1]. Because diseases due to cognitive impairment, such as dementia, cause a large decline in ADL in the beginning, ADL evaluation is an important indicator of whether such cognitive decline is having a great influence on daily living [2]. Patients with cognitive impairment can improve their associated ADL by regularly performing ADL evaluations and repeatedly correcting abnormal behaviors that do not fit a given situation, which are also effective strategies in preventing the worsening of symptoms [3]. For this purpose, regular rehabilitation treatment is required. However, because of the characteristics of these kinds of patients, it would be much more effective to perform it directly at the patient’s home rather than at a hospital. However, for such remote rehabilitation treatment to be possible, a very accurate and precise ADL measurement platform is required. Currently, it is impossible to measure ADL levels indirectly through an interview between patients and their doctors, the method that is mainly used worldwide [4,5]. This new ADL evaluation platform should be able to recognize unit ADL behaviors and accurately and in real time judge various situations that occur while the patient performs the behaviors. It will become possible with the addition of a framework for remote rehabilitation treatment on this platform.

In this paper, in order to overcome the limitations of the existing ADL measurement method mentioned above, first of all, a self-organized IoT platform in which IoT devices autonomously and non-invasively measure a patient’s ADL ability in their daily living place was designed and implemented. Second, a remote rehabilitation treatment concept for enhancing the patient’s ADL ability, we call an “e-coaching framework”, which is a framework in which a doctor remotely gives an instruction in a specific ADL scenario, and the patient’s ability to understand and perform the instruction can be measured on-line and in real time, was additionally developed on top of the self-organized IoT platform.

The ADL measurement scenarios required for the proposed e-coaching framework were implemented as rule knowledge-based systems [6]. For the proposed framework because of the collaboration of numerous sensor data in order for computers to analyze human behavior and the many conditions needed to confirm the success of each stage during ADL evaluation, a rule-based system was needed. Moreover, in terms of user behavior analysis, rule-based systems can make error-free judgments based on accurate facts and rules [7]. Additionally, because of the characteristics of the IoT environment, various sensors may be added or scenarios for ADL evaluation modified, making it easy to flexibly modify the knowledge bases of these objects [8]. Thus, the proposed system may be applied to different users in various IoT platform environments.

This paper is organized as follows. Section 2 provides information concerning previous related studies. Section 3 introduces the “e-coaching framework” and its main functions. Section 4 explains the overall structure and detailed operation sequence of the proposed system. Section 5 introduces the knowledge base for various inferences occurring in the system. Section 6 analyzes the experimental data collected in an implemented test environment. Finally, Section 7 concludes by summarizing this study and discussing the need for further research.

## 2. Related Works

A “smart home” is a residential environment, such as an apartment or house, with wired and wireless communication networks consisting of various IoT devices and a central control unit called a hub [9]. Currently, smart homes can analyze situations in space and recognize their users’ behaviors in real time [10]. These technologies receive environmental information collected by various sensors in space and information on the use of furniture and home appliances and are able to judge situations based on either deep learning or numerous predesignated rules. Moreover, a smart home may implement a customized system by means such as specifying a user using wearable devices or sensors that sense the body information of the user [11,12,13,14] and recognizing the indoor location of a person [15]. This is greatly advantageous, as recognizing in real time an emergency or the health conditions of certain users who lack cognitive or movement skills, such as the elderly and dementia patients, becomes possible [16,17,18]. These technologies related to user ADL monitoring are actively commercialized in similar IoT environments because they are highly accurate in recognizing user behaviors [10].

Currently, various studies are being conducted on services that objectively evaluate users’ conditions through the ADL measurements of patients with cognitive impairment using various sensors and IoT platforms. For instance, one study measured the movement state of users in a living environment and warned them according to the ADL evaluation results [19]. It used infrared motion sensors and door sensors in an IoT platform environment, detected user motion events near the sensors, and reflected these in the ADL evaluations. However, it only detected movements and did not infer what specific actions the user had taken, resulting in an unreliable evaluation of the user’s cognitive functions.

Another research study focused on an integrated system that detects user behavior from sensors placed in the homes of dementia patients and transmits and records the patient’s ADL through cloud services [20]. This allowed the placement of IoT devices and sensors in the spaces of the patient’s daily living environment to detect abnormal behaviors and dangerous situations in real time. However, this study did not consider the system’s scalability against additional ADL scenarios or related sensors, making it difficult to implement a flexible ADL measurement framework.

## 3. E-Coaching Framework

### 3.1. Subsection

**Figure 1:**
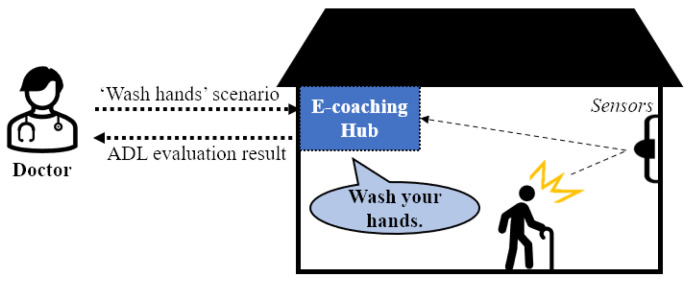
Remote e-coaching framework between a doctor at a hospital and a patient at home.

The basic concept for the remote e-coaching framework is shown in Figure 1. A doctor gives instructions in a scenario for ADL evaluation in conjunction with the hub installed in a patient’s home to evaluate the ADL in the residential environment of the patient with cognitive impairment. The hub assists in real-time remote ADL evaluation by having the patient perform the corresponding ADL evaluation based on the received signal and delivering the evaluation results to the doctor. Therefore, the proposed system should use sensors and an e-coaching hub inside the house to recognize the patient’s behavior and provide coaching services tailored to specific situations.

The user’s daily behavior consists of several unit-behaviors. For example, to do “flush the toilet and turn off the light,” small units of actions such as “enter the bathroom”, “flush the toilet”, “turn off the light”, and “get out of the bathroom” must be performed in order. In this paper, this kind of daily behavior (e.g., “flush the toilet and turn off the light”) is referred to as an “ADL scenario”. Every ADL scenario in the system consists of small goals composed of several stages, and the user must perform each stage accurately, in order, and in time. Each stage that constituted the scenario was named a “step”, and the goal unit-behavior of each step was named the “target behavior”.

**Figure 2:**
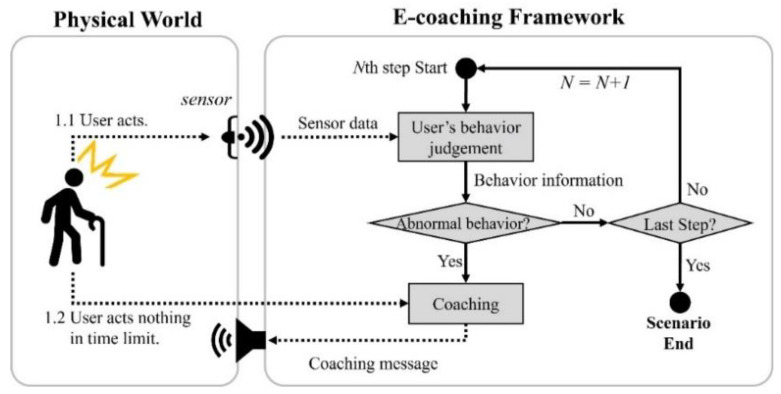
The flow of the ADL evaluation scenario in the proposed framework.

Figure 2 shows a process where a user performs an ADL scenario. When the sensor detects a user’s behavior within the time limit, the sensor data are transmitted to the proposed system. The system analyzes the user’s behavior using the sensor data, compares it with the target behavior of the ongoing step, and performs appropriate coaching or proceeds to the next step (or ends the scenario). If the user does not do anything within the time limit, the system calls for the user’s attention by providing coaching on the step.

### 3.2. Coaching Service

Table 1 shows that when performing an ADL scenario, the situations are largely classified into three categories according to the user’s behavior. In a “normal” situation, where the user performs the target behavior of the corresponding step within the time limit, coaching is not performed. However, in an “abnormal” situation, where the target behavior is not performed, or a “timeout” situation, where no behavior is performed within the time limit, the proposed system provides appropriate coaching to the user.

**Figure 3:**
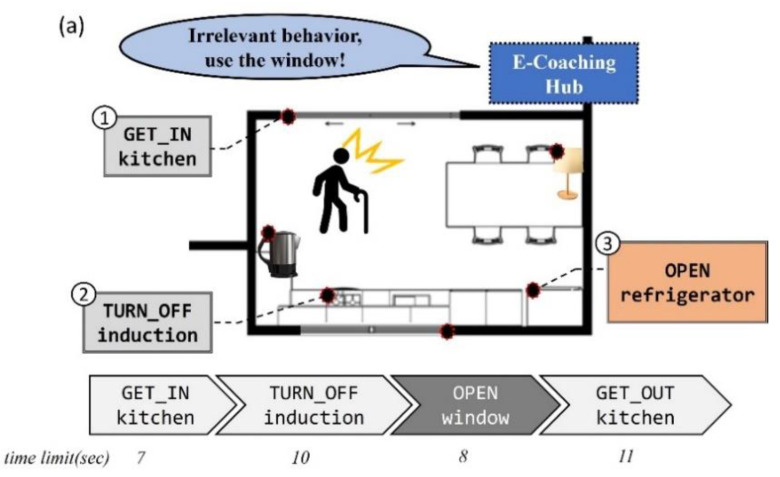

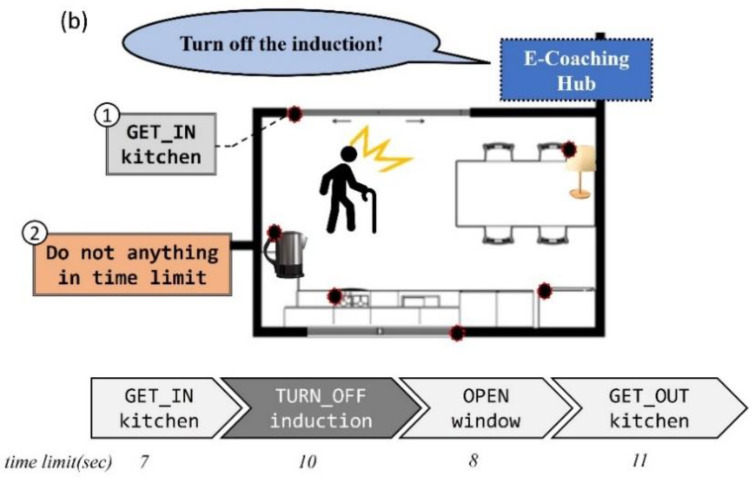
(**a**) Coaching situation caused by an abnormal behavior done in step 3. (**b**) Coaching situation caused by not doing any behavior within the time limit in step 2.

First, in an “abnormal” situation, after the analysis of which part of the user’s behavior does not match the target behavior, coaching is immediately provided so that the correct behavior can be performed. Figure 3a shows an example situation. During the ADL scenario of “turn off the induction and open the window in the kitchen, and get out,” when a behavior not related to the scenario is performed, such as “OPEN refrigerator” rather than “OPEN window” (the target behavior of step 3), the proposed system recognizes that the behavior is not related to the target behavior and provides appropriate coaching for the step. This coaching compares the user’s behavior with the target behavior and provides a coaching message.

Moreover, in a “timeout” situation, when the user does not do anything within the time limit of the step, the proposed system coaches the user on the target behavior of the step. Figure 3b shows an example situation. During the same ADL scenario as in the previous situation, when the user does not perform the target behavior within the time limit in step 2, coaching for the step’s target behavior is provided. In this case, even though the user has received coaching once in the corresponding step, if the timeout situation is repeated thereafter, coaching with the same content is provided again. This e-coaching system was implemented using voice service to allow users to intuitively respond to coaching messages.

**Figure 4:**
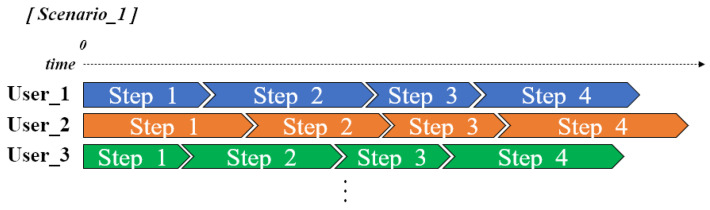
The time limit table required for each user to perform each step in the scenario.

As can be seen from Figure 4, each person has different cognitive and movement abilities; the time taken to perform each step of the ADL scenario during evaluation varies clearly from user to user. Accordingly, in the proposed system, the time limit given for each step was configured differently considering the characteristics of each user. This means that user-tailored ADL scenarios are possible even in places where there are many patients with cognitive impairments, such as in care facilities.

## 4. Architecture of the Proposed System

### 4.1. Entire Structure of the Proposed System

**Figure 5:**
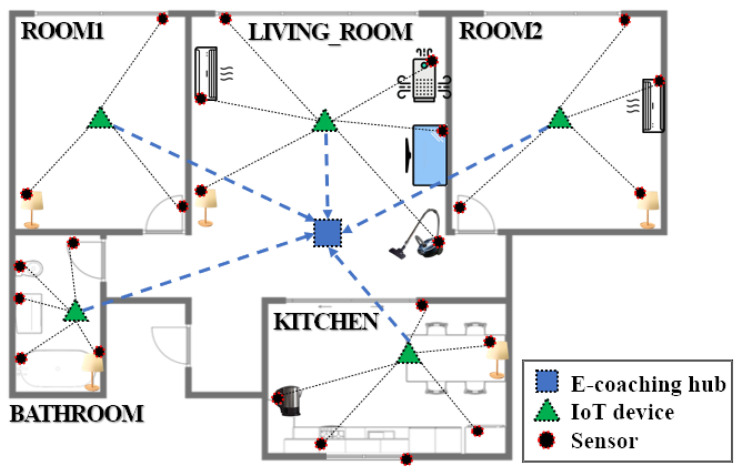
Overall structure of the coaching framework in a smart home.

Figure 5 is an example of the overall appearance of three types of devices (sensor, IoT device, and e-coaching hub) properly placed in a user’s daily living environment. The proposed system was constructed to recognize user’s behavior information in real time using various sensors without the help of an external assistant. This furthers an existing study that required one main hub connected to the sensors to perform designated ADL scenarios [21].

First, “sensor” devices are attached to several home appliances and furniture in the home. When the user uses a home appliance or furniture, sensor data are collected from an “IoT device” that communicates with the sensors which is either wired or wireless. This IoT device is located in each unit-space, such as the living room, kitchen, or toilet, so it can combine the data with its own location information, which allows it to determine the user’s current location and behavior. The “e-coaching hub” is a device that implements the e-coaching framework (a system proposed by this paper) and that is placed in a suitable position for stable communication with all of the IoT devices.

### 4.2. Task Structure of E-Coaching Hub

**Figure 6:**
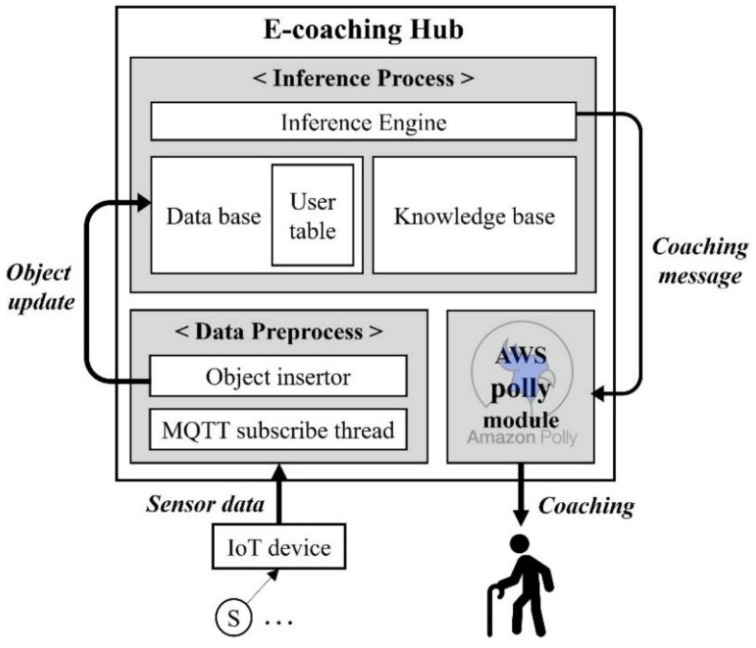
Main processes that compose the proposed system and the main data flows.

Figure 6 shows the main processes and the main data flows constituting the proposed system. First, the “inference process” is responsible for all the inferences that occur during ADL evaluation and is based on using a rule-based system as the nucleus of the coaching framework. The “knowledge base” is composed of rules for various situations that occur during ADL evaluation. The “database” is a set of condition data for executing these rules, such as the data found in all sensor objects and ADL scenario objects, including the “user table,” which contains user dependent information such as step time limits. The “data preprocess” task is responsible for the preprocessing of sensor data and is a preparatory stage for the execution of the inference process. This process consists of an “MQTT subscribe thread” that receives real-time sensor data from the IoT devices and an “object insertor” that extracts important information necessary for inference from the data and updates the information to the corresponding sensor object in the database. Finally, the “AWS Polly module” is a text-to-speech (TTS) service module which receives coaching messages created by the inference engine and provides voice coaching to users.

### 4.3. Detailed Sequence

**Figure 7:**
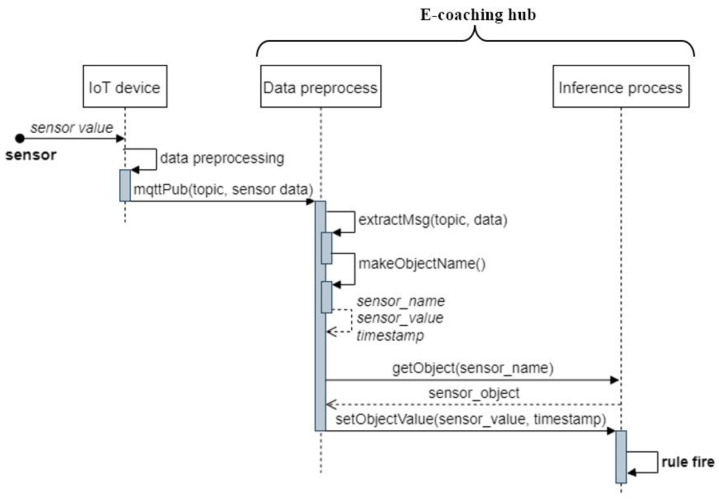
Sequence diagram of the detailed processes for each step of an ADL evaluation scenario.

Figure 7 shows the detailed inference process which occurs in the e-coaching hub. First, the sensors connected to an IoT device detect the user’s behavior, properly preprocess the collected sensor data, and then publish the sensor data message through a designated topic using the MQTT protocol. The topic is of an “environment/sensor_space/sensor_type” format and contains information on the corresponding unit space and the corresponding sensors. For example, the topic “home/room2/window” is related to the window sensor data in room 2. After MQTT subscribes the message, the data preprocess task of the e-coaching hub extracts information on the unit space to which the sensor belongs, the type of sensor, and the value of the sensor to the topic and the message. Afterward, the sensor object is found in the database of the inference process, and the related information is updated. Then, a rule that satisfies the condition fires, and actions related to the rule are executed, such as coaching or updating the database in the inference process.

## 5. Knowledge Base

For all the inference processes that occurred during ADL evaluation, a relevant knowledge base was implemented as a rule-based system software tool called CLIPS (C Language Integrated Productions System) [22]. Currently, about 50 rules are defined in the knowledge base, and addition/reduction is possible at any time. When a rule change occurs, the knowledge base is reloaded in the related programs in the e-coaching hub.

### 5.1. Modularization of Rules

Numerous situations can occur as a user performs each scenario for ADL evaluation. Therefore, implementing rules for all these situations is not only difficult but also inefficient. Thus, this paper modularized the rules for several inferences required in the proposed system in order to solve this problem and to further consider the scalability of users, sensors, and ADL scenarios.

**Figure 8:**
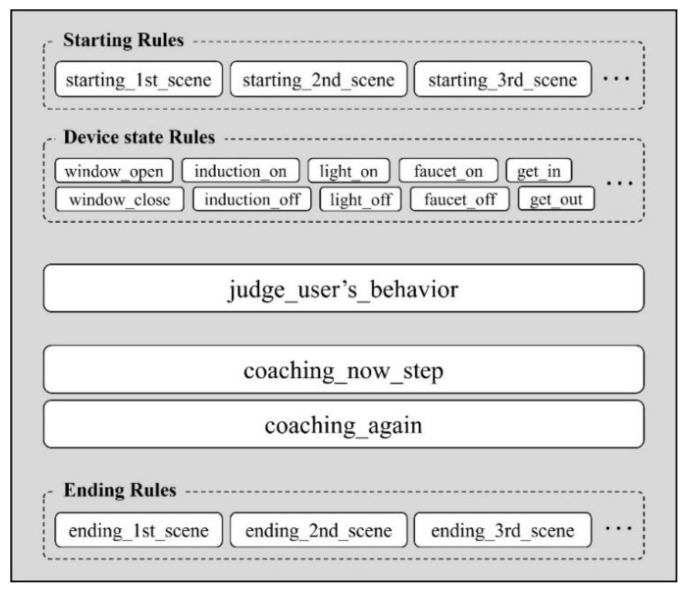
The main components of the knowledge base required for ADL evaluation and the modular rules that are included with them.

Figure 8 shows the modular rules used in the proposed system. First, a starting or ending rule corresponds to each scenario at the start or the end of the ADL scenario. Moreover, “device state rules” determine the state of sensors (e.g., “window open” and “light on”) when detecting a change in the sensor value of each unit space. By recognizing the state change of the sensor using these rules, the system recognizes in real time what the user has done. The “judge_user’s_behavior” rule provides appropriate coaching for the ongoing step based on the user’s behavior information, and the “coaching_now_step” and “coaching_again” rules provide coaching for a timeout situation.

### 5.2. Objects Related to the ADL Scenario

This paper provides ADL scenarios consisting of unit-behaviors that can be sufficiently known only by using the sensor values of IoT devices (e.g., “moving spaces,” “using furniture,” and “using home appliances”) and implements these as objects to be used efficiently in the modular rules described above.

**Figure 9:**
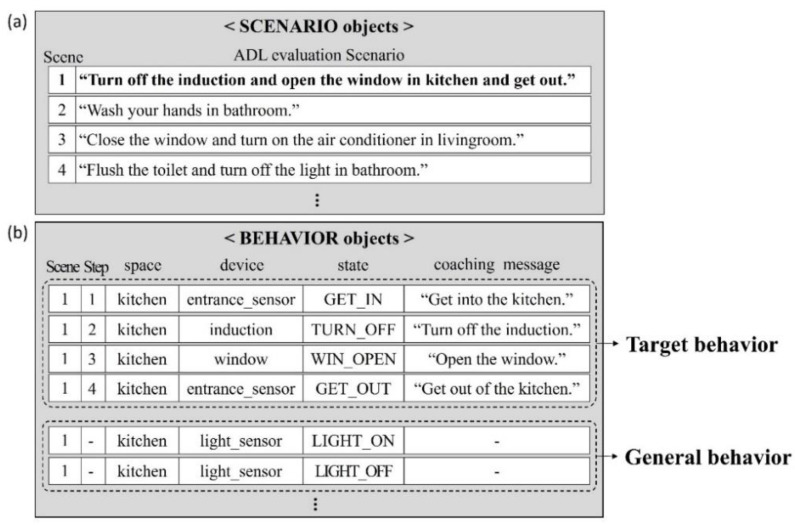
(**a**) ADL evaluation SCENARIO objects. (**b**) BEHAVIOR objects related to Scenario_1.

Figure 9a shows examples of “SCENARIO objects”. These consist of behaviors in daily living that require the use of various tools, such as “turn off the induction and open the window and get out” or “close the window and turn on the air conditioner in the living room”. Furthermore, when performing each ADL scenario, “BEHAVIOR objects” are configured for behaviors generally performed by the user. Each object contains the number of relevant scenarios, the number of steps required for the behavior, the space related to the behavior, the type of sensor responding to the behavior, the current state of the sensor, and the coaching message the system will provide to the user if the behavior is not performed. Figure 9b shows the behavior objects for Scenario_1 in Figure 9a. It consists of four “target behavior” objects to complete Scenario_1 and “general behavior” objects that do not affect the ADL evaluation but are essential unit-behaviors, such as “turn on/off light”.

### 5.3. Starting and Ending Rules for ADL Evaluation Scenarios

**Figure 10:**
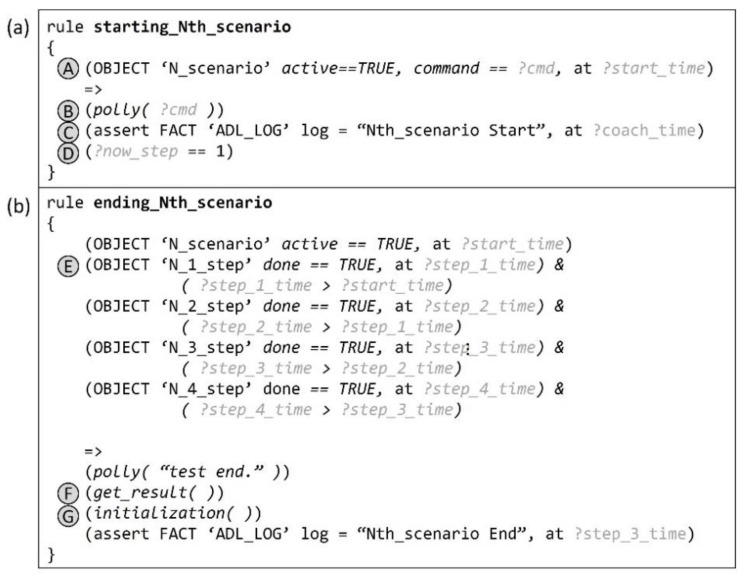
(**a**) Rule for starting an Nth scenario. (**b**) Rule for ending an Nth scenario when the user has performed all steps in the scenario.

Figure 10a briefly shows the rule used to start an Nth ADL scenario. When the proposed system is signaled to start the Nth scenario and (A) the corresponding scenario object is activated, (B) the command for the scenario is delivered to the Polly module and notified to the user by voice. At the same time, (C) an “ADL_LOG” fact indicating the start of the corresponding scenario is generated in the database.

Every ADL scenario consists of several steps. If all steps are performed accurately in order, the ADL evaluation for a scenario ends. Figure 10b shows the ending rule for the Nth ADL scenario. (E) If the condition requiring that all the step objects related to the scenario have been performed in order is satisfied by them being in the “done” state, a history log fact including the end time and a Polly voice message indicating the end of the ADL evaluation are generated. The rule also (F) outputs all ADL_LOGs left during the ADL evaluation and (G) initializes all databases related to the scenario.

### 5.4. Rule for Judging User Behavior

**Figure 11:**
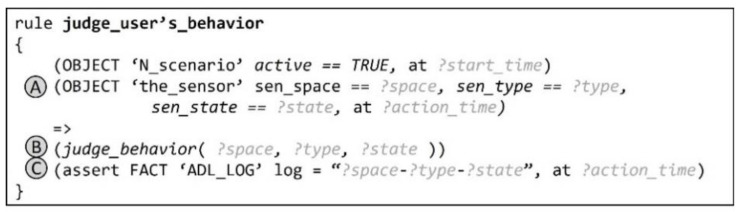
Rule for determining the suitability of a user behavior in an ongoing step.

Figure 11 is a code that briefly shows the rule for determining and processing whether a user’s behavior recognized in real time is appropriate for an ongoing step. (A) When the condition that the sensor object detected in real time be updated has been satisfied, (B) on the basis of the sensor space, sensor type, and sensor state information of the object, the built-in function “judge_behavior” is executed to determine whether the user’s real-time behavior is appropriate for the ongoing step. Moreover, (C) a history log fact about such user behavior is generated.

**Figure 12:**
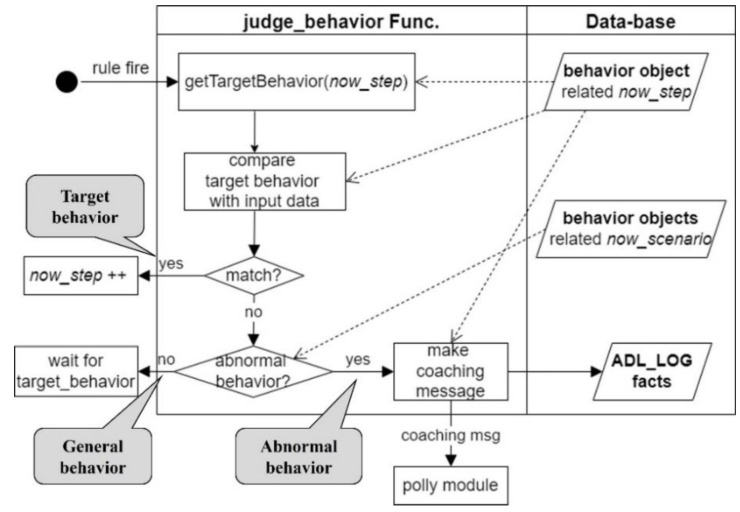
Flowchart showing the process after determining the suitability of the user’s behavior in the “judge_behavior” function.

Figure 12 shows the operation process of the “judge_behavior” built-in function, which determines the suitability of the user’s behavior and is mentioned above. This function approaches the BEHAVIOR object of the current step and compares it with the user’s behavioral information. If it matches, the user is judged to have done the target behavior. Conversely, if it does not match, every behavior related to the scenario is searched to determine whether it is an abnormal behavior. If it is an abnormal behavior, an appropriate coaching message is generated and sent to the Polly module and a coaching history log is left. Otherwise, it is classified as a “general behavior,” and we wait for the next user behavior.

### 5.5. Rule for Coaching by Timeout

When a timeout situation occurs because a user does not act during an ongoing step, the proposed system provides coaching about the step at the appropriate time based on the time limit value of the user table and the CLOCK object representing the current time.

**Figure 13:**
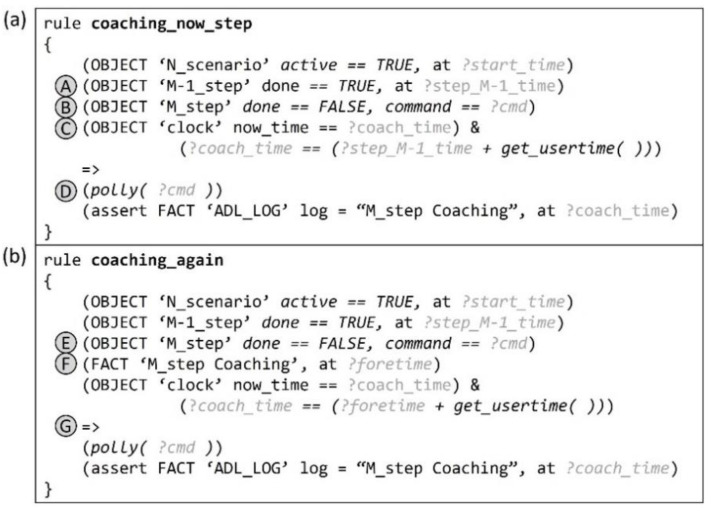
(**a**) Rule for coaching when a user does not do anything even after the time limit for the step has elapsed. (**b**) Rule for determining the situation when the same coaching should be done over the time limit.

Figure 13a shows the rule for the coaching situation when a timeout occurs beyond the time limit for an ongoing step. This case requires that (A) the previous step has been performed and (B) the ongoing step has not been performed. Moreover, (C) the CLOCK object’s value should indicate the time after the time limit from the time when the previous step was performed. After this rule fires, a voice message about the target behavior of the ongoing step is left for coaching, and (D) a history log fact is generated.

Figure 13b is the rule for doing the same coaching when a timeout occurs again after a previous timeout coaching situation. Almost the same conditions as those of the “coaching_now_step” rule are required but with the addition of the following: (E) a history log where the coaching time for the immediately preceding step has been recorded, and (F) the CLOCK object indicating the time after the time limit from the previous coaching time has been added. Thereafter, a (G) coaching history log fact is similarly generated along with a voice message for the target behavior.

## 6. Implementation and Evaluation

**Figure 14:**
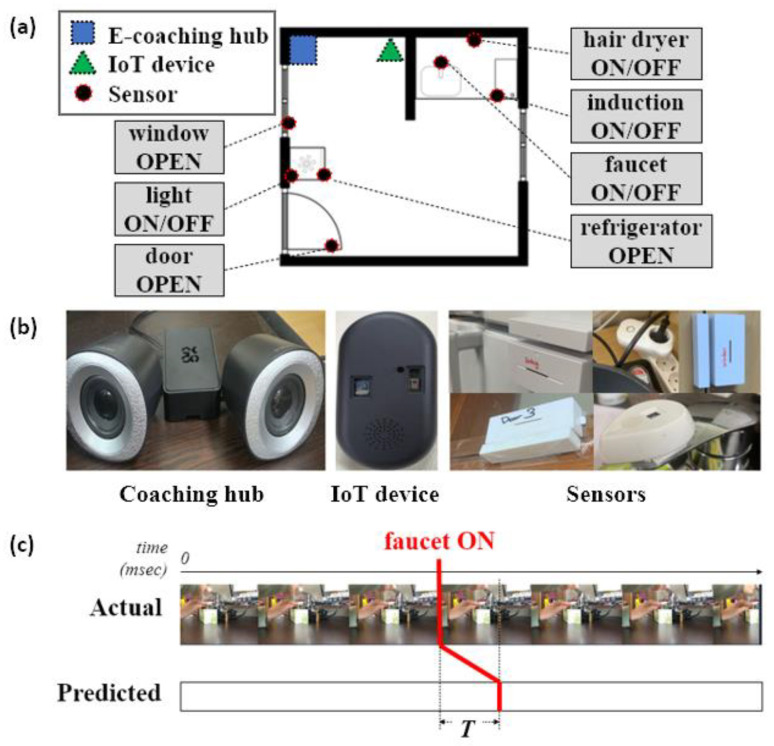
(**a**) Test environment for the progression of an ADL evaluation scenario. (**b**) E-coaching hub, IoT device, and sensors constituting the test environment. (**c**) Measurement of the time gap T between the actual and predicted behaviors by the system.

In this paper, in order to verify the effectiveness of the proposed e-coaching framework and knowledge base system, a testbed in a real environment was constructed and tested, as shown in the Figure 14.

Figure 14a shows the constructed test environment. Figure 14b shows the e-coaching hub, IoT device, and sensors used in the test environment. Figure 14c briefly shows a method for measuring the time it takes for the proposed system to recognize the user’s real-time behavior. A video was taken while a user performed a specific unit-behavior directly, and the difference between the time the behavior was performed in the video and the time the system recognized the behavior was measured as T. The smaller the T value, the more deterministic the system’s detection of a specific situation.

The sensors in this testbed are mobile tags that can be attached to home appliances, and a current sensor to check whether a hairdryer is used has also been installed. The mobile tag has 6-axis acceleration, 3-axis gyro, and ambient light sensors, and has a built-in BLE communication function. An IoT device in a unit space performs BLE bidirectional communication with mobile tags, and an IoT device uses an e-coaching hub and MQTT protocol. In order to minimize the patient’s behavior time error due to the delay rate caused by MQTT messaging, the time measured based on the e-coaching hub time for communication between sensors and the IoT device is regarded as the patient’s execution time. A time stamp for each time value is embedded in the MQTT message and sent as data to the e-coaching hub. Therefore, the effect of the communication delay between the e-coaching hub and the IoT device is not included in the patient behavior measurement time.

### 6.1. Accuracy of the User Behavior Recognition

In performing the ADL scenario, determining how quickly and accurately the proposed system recognizes the user’s behavior is necessary. Thus, the evaluation factors were as follows:

- Does the system accurately recognize the numerous unit-behaviors performed within the time limit?
- How long does it take for the system to recognize a unit-behavior?

Eleven unit-behaviors were able to be performed while using the objects in the environment in Figure 14a. Each unit-behavior was performed several times to confirm that the system had recognized the behavior accurately. Furthermore, *T_act*, the time it took for the system to recognize each unit-behavior, was obtained following the method for obtaining *T* in Figure 14c. Its overall average value, “*T_(act.avg)*”, was also obtained.

(1) $$T_{act}=\frac{\sum \left(T_{unit\;behavior}\right)}{\left(the\;number\;of\;unit\;behavior\right)}$$

(2) $$T_{act.avg}=\frac{\sum \left(T_{act}\right)}{\left(total\;number\right)}$$

Table 2 categorizes the measurement results of the behavioral perception of the system into three categories after the performance evaluation and shows the ratio for each item and *T_(act.avg)*. Each unit-behavior was performed 30 times—for a total of 330 times—to obtain accurate results. The time limit for each behavior was arbitrarily set to 3 s.

A total of 309 times, actions were accurately recognized, and the accuracy was 93.6%. The percentage of behavior recognition failure due to defective sensors or problems in the data transmission process was 3.9%. The ratio of behavior non-recognition within the time limit was 2.5%. Moreover, the time *T_act* was measured as 416ms.

**Figure 15:**
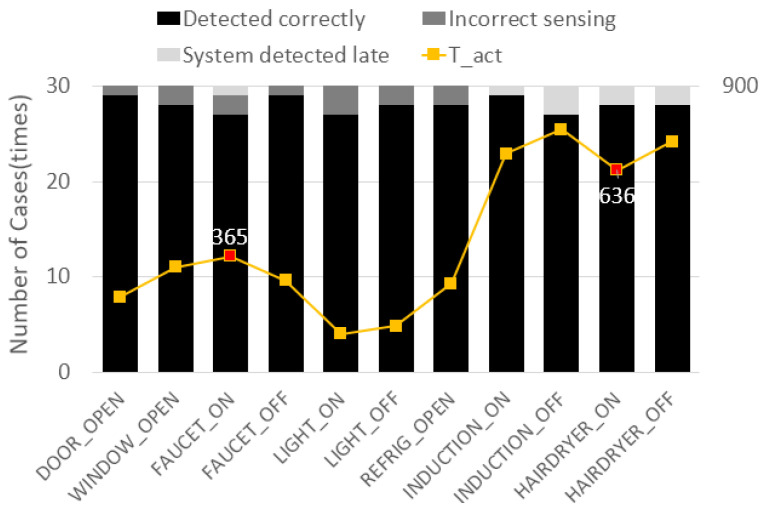
Detailed user behavior judging the results and the average value of the time gap $T_{act}$.

Figure 15 shows the results for the behavioral cognitive measurements for each behavior. In this graph, the unit-behaviors related to “induction” and “hair dryer” have larger *T_act* values than those of the other behaviors. The sensors connected to the induction and the hair dryer used in the performance evaluation measure the power consumption, so they recognize that they are in a “TURN_ON” or “TURN OFF” state when the power value gradually changes and reaches a certain value. Therefore, the proposed system recognizes the ON/OFF state of home appliances relatively slowly. Thus, it is confirmed that the accuracy levels for these behaviors were less than those for the other sensors simply because they exceeded the time limit.

### 6.2. Measurement of Coaching Timing

The proposed system should not only recognize the user ADL, but also be able to provide quick and appropriate coaching services depending on the situation. Thus, the coaching service performance was evaluated by appropriately placing abnormal situations that may occur during ADL evaluation in each step of the scenario in an IoT test environment such as that in the previous performance evaluation.

**Figure 16:**
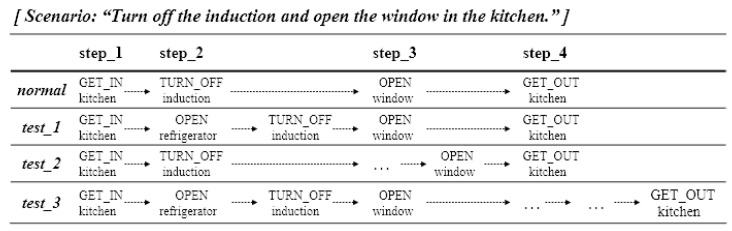
Test performance scenario mixed with several abnormal behaviors.

The first line in Figure 16 is a routine when the scenario is normally performed, and test_1 is a routine that is immediately coached upon performing an abnormal behavior within the time limit in step_2. Test_2 is a routine to receive coaching upon timeout in step_3, and test_3 is a routine when coaching is performed after an abnormal behavior in step_2, with coaching given upon every timeout in step_4. Defining several time elements to be measured in each routine is necessary for determining the accuracy of the time needed for providing coaching services in these three test routines.

**Figure 17:**
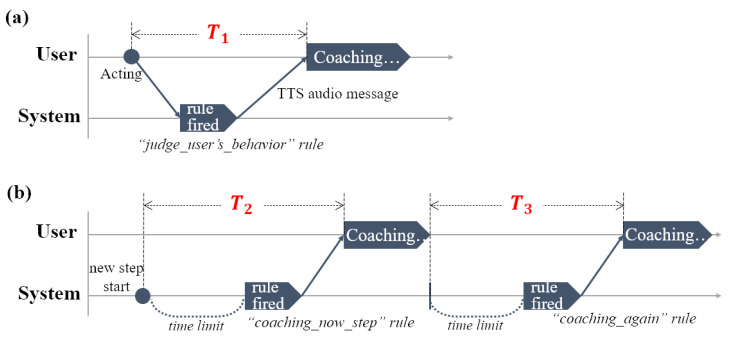
Coaching situations for the rules (**a**) “judge_user’s_behavior” (**b**) “coaching_now_step” and “coaching_again”.

Figure 17a shows the immediate coaching routine when a user’s real-time behavior does not match the target behavior of the step. T_1 is the difference between the time the user acts and the time the user starts to hear coaching for their behavior. Figure 17b shows the test routine corresponding to a timeout. T_2 is the difference between the time a history log notifies the start of the step and the time the user starts to hear coaching. T_3 is the difference between the time when the previous coaching ended and the time the user starts to hear the same coaching again.

The evaluation factors are as follows:

- When the user performs an abnormal behavior within the time limit, is coaching provided immediately?

$$T_{1}$$

- Is coaching provided as soon as the time limit for the ongoing step is reached?

$$T_{2}-\mathrm{time}\;\mathrm{limit},\;T_{3}-\mathrm{time}\;\mathrm{limit}$$

Table 3 shows the results from the test routine for the above evaluation factors. Three test scenarios were performed thirty times each, and the average value for the times measured can be checked for each test routine. Five results found from the three test routines included approximately 495ms as the time it took to send a coaching message that was produced as a result of the rule being fired to an external Polly server and returning as a voice message through the TTS service. These five results represent very small time values, except for the time it took to convert the coaching message into a voice message or to receive the sensor value from the IoT device. Given the above results, the coaching service during the ADL evaluation was deterministic for the user’s abnormal behavior or timeout situation.

## 7. Conclusions

The remote e-coaching framework proposed in this paper enabled real-time ADL evaluation with the help of an external doctor so that a patient with cognitive impairment could perform rehabilitation treatment in a residential environment equipped with various sensors. It can thus be applied to patient rehabilitation by being used to provide real-time coaching services for abnormal situations, furthering several existing studies that recognized or evaluated user behavior using sensor data. It is a more convenient and objective approach than evaluating ADL within existing medical facilities, as the use indirect methods, such as taking personal videos and doing evaluations with a close caregiver. Additionally, this system can be applied not only to a user’s home but also to care facilities which have many patients with similar diseases. The proposed system showed high accuracy in the area of behavioral cognition or coaching services using a rule-based system. However, its determination of ADLs is restricted by the limited use of sensors. Moreover, even if numerous different types of sensors were used in IoT platform environments, there would still be difficulties in accurately recognizing all the users’ behaviors using only sensors. These problems can be sufficiently solved by deep learning based on various sound data or real-time change values in the environmental data that occur during daily activities. In these cases, more diverse ADLs will be recognized, and practical ADL evaluation scenarios can be constructed and implemented.

We hope that the proposed e-coaching framework can aid patients with cognitive impairment in living stable lives in their homes with the help of IoT devices.

## Figures and Tables

**Table 1:** Cases according to a user’s behavior at each step of the ADL scenario.

	Act Within the Time-Limit of Each Step?	Is the Behavior Consistent with the Target Behavior?	Need Coaching
Normal	O	O	**X**
Abnormal	O	X	**O**
Timeout	X	-	**O**

**Table 2:** Comprehensive cognitive performance results for all unit-behaviors.

Total (Times)	Accurately Recognized (Times)	$T_{act.avg}\;(\mathrm{ms})$	Ratio of Incorrect Sensing (%)	Ratio of Behavior Non-recognition within the Time Limit (%)	Accuracy (%)
330	309	416	3.9	2.5	93.6

**Table 3:** Performance evaluation results for the coaching timing measurement.

	Execution (Times)	$T_{1}\;(\mathrm{ms})$	$T_{2}-Time\;Limit\;(\mathrm{ms})$	$T_{3}-Time\;Limit\;(\mathrm{ms})$
test_1	30	644	-	-
test_2	30	-	537	-
test_3	30	631	526	582

## Data Availability

Not applicable.
